# The Association of Current Violence from Adult Family Members with Adolescent Bullying Involvement and Suicidal Feelings

**DOI:** 10.1371/journal.pone.0163707

**Published:** 2016-10-06

**Authors:** Shinya Fujikawa, Shuntaro Ando, Shinji Shimodera, Shinsuke Koike, Satoshi Usami, Rie Toriyama, Sho Kanata, Tsukasa Sasaki, Kiyoto Kasai, Yuji Okazaki, Atsushi Nishida

**Affiliations:** 1 Department of Neuropsychiatry, Graduate School of Medicine, The University of Tokyo, Tokyo, Japan; 2 Department of Psychiatry & Behavioral Science, Tokyo Metropolitan Institute of Medical Science, Tokyo, Japan; 3 Department of Neuropsychiatry, Kochi Medical School, Kochi University, Kochi, Japan; 4 University of Tokyo Institute for Diversity & Adaptation of Human Mind (UTIDAHM), Tokyo, Japan; 5 Center for Evolutionary Cognitive Sciences, Graduate School of Art and Sciences, The University of Tokyo, Tokyo, Japan; 6 Faculty of Human Sciences, Division of Psychology, University of Tsukuba, Ibaraki, Japan; 7 Department of Psychiatry, Teikyo University School of Medicine, Tokyo, Japan; 8 Department of Physical and Health Education, Graduate School of Education and Division for Counseling and Support, The University of Tokyo, Tokyo, Japan; 9 Department of Psychiatry, Tokyo Metropolitan Matsuzawa Hospital, Tokyo, Japan; Shinshu University School of Medicine, JAPAN

## Abstract

Although several studies have reported that child physical abuse increased the risk for bullying involvement, the effect of current violence from adult family members (CVA) on bullying involvement and suicidal feelings among adolescents has not been sufficiently examined. This study investigated the association of CVA with adolescent bullying involvement and the interaction effect of CVA and bullying involvement on suicidal feelings. This cross-sectional study used data from a school-based survey with a general population of adolescents (grades 7 to 12). Data were collected using a self-report questionnaire completed by 17,530 students. Logistic regression analyses were performed to explore the association of CVA with adolescent bullying involvement and suicidal feelings. The overall response rate was 90.2%. The odds of students being characterized as bullies, victims, and bully-victims were higher among adolescents with CVA than without CVA (odds ratios (OR) = 2.9, 95% confidence interval (CI), [2.3–3.7], 4.6 [3.6–5.8], and 5.8 [4.4–7.6], respectively). Both CVA (OR = 3.4 [95% CI 2.7–4.3]) and bullying (bullies, victims, and bully-victims; OR = 2.0 [95% CI 1.6–2.6], 4.0 [3.1–5.1], 4.1 [3.0–5.6], respectively), were associated with increased odds of current suicidal feelings after adjusting for confounding factors. Furthermore, positive additive effects of CVA and all three types of bullying involvement on suicidal feelings were found. For example, bully-victims with CVA had about 19-fold higher odds of suicidal feelings compared with uninvolved adolescents without CVA. This study, although correlational, suggested that CVA avoidance might prevent bullying involvement and suicidal feelings in adolescents.

## Introduction

Bullying involvement is defined as follows: “a child is *being bullied* when another child, or a group of children, say or do nasty and unpleasant things to him or her. It is also bullying when a child is teased repeatedly in a way he or she does not like [1].” Bullying involvement, including bullies (who only bully others), victims (who are only bullied by others), and bully-victims (who both bully others and are bullied by others), is a global problem that involves approximately 30% of adolescents across multiple countries [2]. Bullying is also a serious public health issue in adolescence as it can cause internalizing problems [3], depression [4–6], and suicidality (including suicidal feelings and attempts) [5,7–11]. Furthermore, the effect of bullying victimization on mental health problems can last as long as forty years [12,13]. Therefore, examining the risk factors for bullying involvement is required. While the typical focus of research and intervention on bullying involvement has been on schools [14,15], the importance of family interventions has also recently been suggested [12,16]. Several studies have revealed family-related factors in bullying involvement, including maladaptive parenting such as hostility and threat [17], overprotection [17], and maternal mental health [18]. In addition, many studies have indicated that family violence in childhood, such as child abuse and domestic violence [16,19], is associated with bullying involvement. Bowes and colleagues reported that child abuse predicts victims and bully-victims and that domestic violence predicts bullies [16].

Negative parent-child relationships such as family violence were considered an origin source for traditional bullying [20]. Family violence includes any violence from adult cohabitants regardless of its purpose including a means of discipline. Children may imitate family violence and bully peers. On the other hand, children may decrease self-esteem from experiencing family violence and be bullied by peers. Even though there are young people who suffer from violence from family members in adolescence and such violence could be a target of intervention, current violence from adult family members (CVA) experienced by adolescents has not been sufficiently examined in terms of risk for bullying involvement. In the single previous study that reported the association between CVA, bullies, victims, and bully-victims [21], the overall response rate was relatively low (middle school students = 55.8%, high school students = 66.7%). Further, the study did not examine interaction effects between CVA and bullying involvement on adolescents’ mental health. To date, no study has examined the association of CVA with adolescent bullying involvement and the interaction effects of CVA and bullying involvement on suicidal feelings simultaneously. We hypothesized that CVA would be associated with increased odds of bullying involvement and of suicidal feelings among adolescents.

This study, therefore, examined whether CVA was associated with bullying involvement and how CVA affected the relationship between bullying involvement and suicidal feelings using a large community population sample of adolescents.

## Methods

### Study Design, Sample, and Survey Procedures

This study used data from a multi-center cross-sectional survey of the general population of adolescent students in public junior high schools (7th–9th grades) and high schools (10th–12th grades) in Japan [22–24]. We examined the general psychopathologies and adolescents’ lifestyles in 2008 and 2009. Although high school education is not compulsory under Japanese law, approximately 98% of adolescents enter high schools [25]. The principal study investigators asked for survey participation from the head and administrators of every public junior high schools in Tsu city (population = 280,000) of Mie prefecture and public junior high and high schools in Kochi prefecture (population = 790,000). Tsu city is a medium-sized Japanese city and Kochi prefecture consists of both rural and urban areas [26]. Subsequently, the administrators and heads of the schools consulted with teachers and parents about survey participation. Guidelines for the distribution and collection of the self-report questionnaires were provided to the teachers. After obtained written informed consent from the parents, teachers distributed the self-report questionnaires to students along with envelopes to seal their completed questionnaires in each school. In order to obtain reliable responses from students, all responses were made anonymously and teachers explained that study participation was voluntary, confidential, and carried no disadvantage for non-participation. Teachers reported the total number of absences and participants on the day of the survey. The research team received the sealed envelopes from each school. This study was approved by the ethics committees of the Tokyo Metropolitan Institute of Psychiatry (since 2011, Tokyo Metropolitan Institute of Medical Science), Mie University School of Medicine, and Kochi Medical School. It was conducted consistent with the Declaration of Helsinki as revised in 1989.

### Participants

Thirteen out of 20 public junior high schools in Tsu city participated in this study; in Kochi prefecture, 32 out of 118 public junior high schools and 28 out of 36 public high schools participated. Of the 19,436 total students, 18,638 from the participating schools were approached at the school (798 or 4.1% were absent); 18,250 (93.9%) agreed to participate in this study. Among the consenting students, data from 720 students were excluded from analyses due to incomplete answers on the any of the variables. Therefore, 17,530 responses (overall response rate = 90.2%) were suitable for analyses (mean age = 15.2 years, SD = 1.7; boys = 49.7%).

### Measures

We used the following items in the self-report questionnaire: CVA, the experience of bullying others or being bullied, current suicidal feelings, alcohol and illegal drug use, and demographic characteristics. Considering the limited time for the survey conducted in school, we used a simple and short question to identify the existence of CVA, bullying involvement, sucidal feelings, and other confounders.

### CVA

The question about CVA was as follows, “Have you suffered from violence by adult cohabitants during the past month?” The participants were asked to choose “yes” or “no.” We regarded the adult cohabitants as their adult family members since most of the adult cohabitants consisted of adult family members. Adolescents who replied positively were defined as those who experienced CVA. Since CVA had no gold standard measure, the original measure of CVA was used in this study. The timeframe of this question was within one month, because we aimed to focus on current violence from adults, but not on childhood abuse. We could not include a question about history of childhood abuse, because the focus of the survey was on the general psychopathologies and lifestyles of adolescents.

Since the scope of this study was to examine whether CVA was associated with bullying involvement or not, and not to investigate a dose-response relationship between them, a single item was used for CVA assessment.

#### Bullying involvement

Two questions about bullying involvement as defined in previous studies [22,23] were included in the questionnaire: “Have you been bullied during the past year?” and “Have you bullied others during the past year?” The participants were asked to choose “yes” or “no” to each question. Since bullying can occur intermittently and be repeated over time [12], we used the timeframe of one year rather than one month to avoid underreporting of bullying. Based on the responses, the participants were classified into the following four groups: 1) uninvolved (those who had not bullied others and had not been victimized); 2) bullies (those who had bullied others, but had not been bullied); 3) victims (those who had been bullied and had not bullied others); and 4) bully-victims (those who had both bullied others and been bullied).

#### Current suicidal feelings

Current suicidal feelings were assessed by the following question, “Do you currently have thoughts that your life is no longer worth living?”[27] The four response choices were “no,” “probably no,” “probably yes,” and “yes.” In order to represent apparent suicidal feelings, “yes” was categorized as the presence of current suicidal feelings as defined in a previous study [28].

#### Other variables

Since bullying involvement has been associated with alcohol use [21,29], drug use [21], and living status [30], we included those variables in the questionnaire as confounding factors. Alcohol use in the past month was assessed by a dichotomous question answered either “yes” or “no.” Additionally, lifetime experience of any drug use was assessed, using the following question, “Have you ever used any illegal drugs?” The response choices were “no,” “only once,” “twice,” and “more than two times.” We defined those who indicated the use of illegal drugs at least once as drug users. Using a question about current cohabitants, living status was categorized into three groups: not living with parents, living with a single parent, and living with both parents. Demographic characteristics, including age, sex, and education level (junior high school/high school) were also assessed.

### Statistical Analyses

To explore the association of CVA with adolescent bullying involvement, multinomial logistic regression analyses were conducted using one crude and two adjusted models (one model adjusting for demographics and another model adjusting for demographics and other confounders). In the logistic regression models, CVA was used as the independent variable and the three types of bullying involvement were used as dependent variables. To assess age-related and sex difference in the association between CVA and adolescent bullying involvement, the interactive effects of CVA and age/sex on bullying involvement were examined using the model adjusting for all confounders. Furthermore, sex-specific associations of CVA and bullying involvement were examined. Next, a crude and an adjusted model were used in binominal logistic regression analyses. In the analyses, CVA and bullying involvement were used as independent variables and current suicidal feelings was used as the dependent variable. Then, students were divided into eight groups according to CVA experience and bullying involvement: 1) uninvolved in bullying without CVA experience; 2) uninvolved in bullying with CVA experience; 3) bullies without CVA experience; 4) bullies with CVA experience; 5) victims without CVA experience; 6) victims with CVA experience; 7) bully-victims without CVA experience; 8) bully-victims with CVA experience. Finally, to examine the risk for current suicidal feelings in each group compared with Group 1, binominal logistic regression analyses were performed using a crude and an adjusted model. Multivariate logistic regression analyses were performed to examine the main effects and any interactive effects of CVA and bullying involvement on current suicidal feelings. All statistical analyses were completed using SPSS statistics version 21 (IBM Corp, New York, USA).

## Results

### Prevalence of CVA, Adolescent Bullying Involvement, and Current Suicidal Feelings

The demographic characteristics of participants as well as the CVA prevalence, bullying involvement (uninvolved, bullies, victims, and bully-victims), and current suicidal feelings are shown in Table 1. Approximately 15% of the participants were involved in bullying. More students reported experiences of bullying others than students who reported experiences of victimization. The majority of students did not experience CVA. About 4% of students reported current suicidal feelings.

**Table 1:** Prevalence of current violence from adult family members, bullying involvement, suicidal feelings, and demographic characteristics.

Category	*N*	%
**Education level**		
**Junior high school**	8,318	47.5
**High school**	9,212	52.5
**Sex**		
**Male**	8,715	49.7
**Female**	8,815	50.3
**Bullying involvement**		
**Uninvolved**	14,902	85.0
**Bullies**	1,301	7.4
**Victims**	832	4.7
**Bully-victims**	495	2.8
**Current violence from adult family members**		
**Violence**	654	3.7
**No violence**	16,876	96.3
**Current suicidal feelings**		
**Yes**	706	4.0
**No**	16,824	96.0

### The Association between CVA and Adolescent Bullying Involvement

The association between CVA and adolescent bullying is shown in Table 2. CVA was associated with increased odds of all three types of bullying involvement (bullies, victims, and bully-victims; all *p* < .001). The associations remained significant even after adjusting for confounding factors.

**Table 2:** The odds ratios for current violence from adult family members with adolescent bullying involvement^a^.

	Bullies		Victims		Bully-victims	
	OR (95% CI)	*p*-value	OR (95% CI)	*p*-value	OR (95% CI)	*p*-value
CVA						
Crude OR	3.85 (3.09–4.80)	<.001	5.53 (4.38–6.99)	<.001	8.04 (6.21–10.42)	<.001
Adjusted OR						
Model 1^b^	3.24 (2.58–4.06)	<.001	4.73 (3.73–6.00)	<.001	6.53 (5.00–8.52)	<.001
Model 2^c^	2.91 (2.31–3.67)	<.001	4.58 (3.61–5.81)	<.001	5.76 (4.39–7.55)	<.001

Abbreviations: CVA: current violence from adult family members; CI: confidence interval; OR: odds ratio;

^a^ Participants uninvolved in any bullying were used as reference

^b^ Adjusted for age and sex

^c^ Adjusted for age, sex, alcohol use, illegal drug use, and living status

### Age-Related and Sex Differences in the Association between CVA and Adolescent Bullying Involvement

Significant interactive effects of CVA and age on all three types of bullying involvement were revealed (bullies: adjusted OR = 1.17 [95% CI: 1.01–1.37], *p* = .04, victims: adjusted OR = 1.18 [95% CI: 1.02–1.38], *p* = .03; bully-victims: adjusted OR = 1.29 [95% CI 1.07–1.54], *p* = .006). The effect of CVA on bullying involvement increased as age increased. A significant interactive effect of CVA and sex on bully-victims was revealed (*p* < .001). Boys with CVA experience had higher odds of being bully-victims than girls with CVA did (boys: adjusted OR = 8.71 [95% CI: 6.23–12.17], *p* = .04, girls: adjusted OR = 2.75 [95% CI: 1.64–4.62], *p* < .001). There were no interactive effects of CVA and sex on bullies and victims.

### The Association between CVA/Adolescent Bullying Involvement and Current Suicidal Feelings

Both CVA (adjusted odds ratio [OR] = 3.39 [95% CI 2.65–4.34], *p* < .001) and bullying involvement (bullies: adjusted OR = 2.02 [95% CI 1.56–2.62], *p* < .001; victims: adjusted OR = 3.98 [95% CI 3.13–5.07], *p* < .001; and bully-victims: adjusted OR = 4.09 [95% CI 3.01–5.57], *p* < .001) were associated with increased odds of current suicidal feelings after adjusting for confounding factors. There was no interaction between CVA and adolescent bullying involvement on current suicidal feelings.

**Fig 1:**
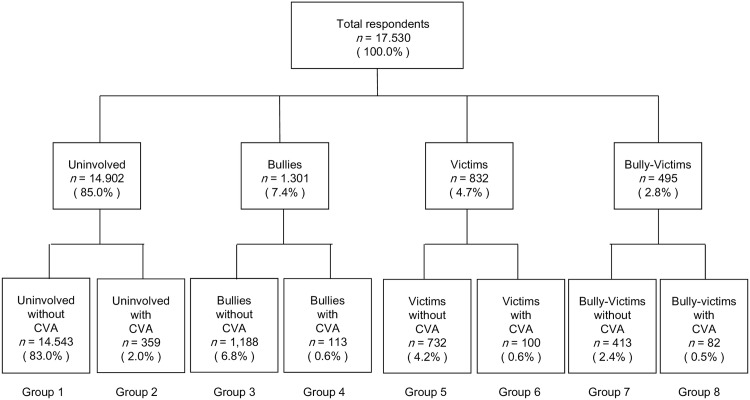
Four clusters and eight groups defined by bullying and current violence from adult family members. Abbreviation: CVA; current violence from adult family members; Group 1) uninvolved in bullying without CVA; Group 2) uninvolved in bullying with CVA; Group 3) bullies without CVA; Group 4) bullies with CVA; Group 5) victims without CVA; Group 6) victims with CVA; Group 7) bully-victims without CVA; Group 8) bully-victims with CVA.

**Fig 2:**
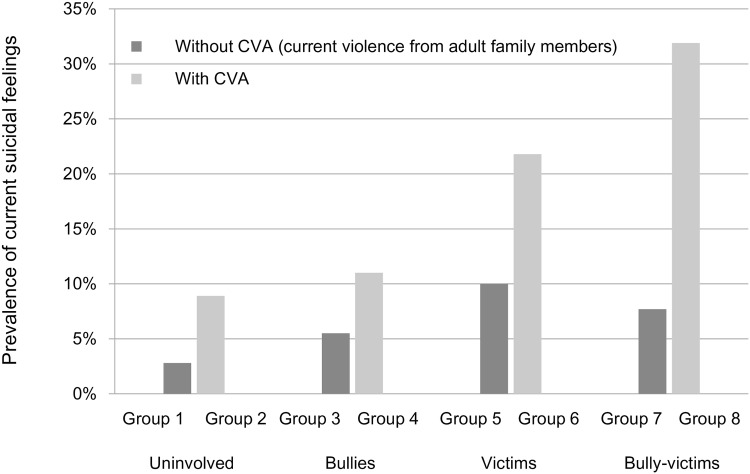
Relationship between suicidal feelings and bullying involvement with current violence from adult family members. Group 1) uninvolved in bullying without CVA; Group 2) uninvolved in bullying with CVA; Group 3) bullies without CVA; Group 4) bullies with CVA; Group 5) victims without CVA; Group 6) victims with CVA; Group 7) bully-victims without CVA; Group 8) bully-victims with CVA.

The distribution of participants in the eight groups divided by the combination of CVA experience and bullying involvement is shown in Fig 1. When comparing the eight groups according to CVA experience and all types of bullying involvement, the prevalence of current suicidal feelings was higher in students with CVA experience than in those without CVA experience (Fig 2). The prevalence was highest in Group 8 (bully-victims with CVA experience, 31.9%).

Compared with Group 1 (those uninvolved in bullying without CVA experience), the other seven groups had increased odds of current suicidal feelings (Table 3). Group 8 (bully-victims with CVA experience) had about 19-fold higher risk for current suicidal feelings when compared with Group 1.

**Table 3:** The odds ratio of suicidal feelings in each group defined by bullying and current violence from adult family members.

	Condition in each group		Current suicidal feelings			
	CVA	Bullying involvement	Crude model		Adjusted model ^a^	
			OR (95% CI)	*p*-value	OR (95% CI)	*p*-value
**Group 1**	No	Uninvolved	Reference		Reference	
**Group 2**	Yes	Uninvolved	3.66 (2.56–5.23)	<.001	3.69 (2.56–5.32)	<.001
**Group 3**	No	Bullies	1.87 (1.43–2.45)	<.001	2.18 (1.65–2.88)	<.001
**Group 4**	Yes	Bullies	5.02 (2.89–8.72)	<.001	5.15 (2.89–9.17)	<.001
**Group 5**	No	Victims	3.80 (2.94–4.91)	<.001	4.21 (3.24–5.47)	<.001
**Group 6**	Yes	Victims	9.80 (6.09–15.77)	<.001	11.34 (6.97–18.44)	<.001
**Group 7**	No	Bully-victims	2.94 (2.05–4.24)	<.001	3.56 (2.45–5.17)	<.001
**Group 8**	Yes	Bully-victims	17.02 (10.68–27.13)	<.001	19.14 (11.53–31.78)	<.001

Abbreviations: CVA: Current violence from adult family members; CI: confidence interval; OR: odds ratio;

^a^ Adjusted for age, sex, alcohol use, illegal drug use, and living status

## Discussion

CVA was associated with increased odds of all three types of bullying involvement (bullies, victims, and bully-victims) among general adolescents. Older adolescents with CVA experience had higher odds of all three types of bullying involvement than younger adolescents with CVA. Boys with CVA experience had higher odds of being bully-victims than girls with CVA did. This study also revealed the positive additive effect of CVA and all three types of bullying involvement on suicidal feelings among adolescents. No interaction between CVA and bullying involvement on current suicidal feelings was observed. To the best of our knowledge, this was the first study that simultaneously revealed the association of CVA with bullying involvement and current suicidal feelings among general adolescents.

CVA was associated with increased odds of bullying involvement in general adolescents, which was consistent with a previous study [21]. There may be several explanations for the association between CVA and increased bullying others. First, adolescents may imitate the violent behaviors of parents at home, which is consistent with social learning theory [31], revealing that behavior can be acquired through direct experience or by observing the behavior of others [31]. Second, the exposure to violence from parents might prevent the development of adolescents’ social problem solving skills, which was associated with aggressive behaviors towards peers [32]. Third, gene-environment interplay may underlie the association. Adolescents who inherits aggressive temperament from their parents may induce hostile actions from peers and be prone to aggressive behaviors [33,34]. Aggressive parents might tend to use CVA, and similarly aggressive children might tend to bully others. With respect to the association between CVA and increased numbers of bullying victims, there may also be several explanations. First, adolescents exposed to violence from parents tend to experience psychological distress and may feel powerless and less confident. They may be more vulnerable to violence and bullying. Second, similar to the reasons proposed for bullies, attenuated assertion and social problem solving skills could lead to being bullied. Third, since bullying victimization was more common in the physically weak and frail [35], adolescents who are victims of violent adult family members at home and of other students in schools may be physically vulnerable. Bully-victims increased by CVA might stem from a mixture of the reasons for bullying and/or being bullied. This might be why bully-victims have the highest risk of adjustment problems.

CVA was associated with increased odds of current suicidal feelings in general adolescents. This result is consistent with previous research showing a relationship between CVA and suicidal ideation [36], which was a more severe stage of suicidal feelings [27]. Consistent with previous studies [7,37,38], all three types of bullying involvement were associated with current suicidal feelings. Especially, bully-victims were at the highest risk of suicidal feelings. This may be because bully-victim status is typically developed over time, and such individuals may be more likely to become suicidal. Furthermore, the positive additive effects of CVA and all three types of bullying involvement on current suicidal feelings were revealed. This may mean that CVA did not develop adolescents’ resilience to aggression from others or it did not make adolescents more vulnerable to the effect of aggression. It should be noted that CVA was associated with not only increased odds of bullying involvement, but also increased odds of suicidal feelings.

There were several strengths of this study. First, this study used a large sample of the general population of adolescents and the overall response rate was very high (90.2%). Second, as the students could respond to the questionnaire anonymously and used envelopes to seal their own completed questionnaires, the underestimate of responses could be avoided to some extent. Third, since we examined not only being bullied, but also bullying others, we revealed the prevalence of all three types of bullying involvement (bullies, victims, and bully-victims), and the associations of all three types of bullying involvement with CVA and current suicidal feelings. Consequently, we suggest that bullying involvement, particularly bully-victims with CVA experience, had a higher risk for current suicidal feelings.

This study had several limitations. First, as this study was a cross-sectional survey, chronological and causal relationships between CVA, bullying involvement, and suicidal feelings could not be determined. In the future, a longitudinal study is needed to clarify if a causal relationship exists. Second, we did not assess past violence from adult family members in childhood, intelligence level, or socioeconomic status, which could be potential confounding factors. Particularly, a history of child abuse may be associated with CVA. Third, the primary measurements (e.g., CVA and bullying involvement) of this study consisted of simple, binary questions. Although we could attain a high response rate using those simple questions, information on frequency and severity were not obtained. Further, the use of such simple questions did not enable the analyses on dose-response relationships between CVA, bullying involvement, and suicidal feelings. Fourth, the participants were limited to the students in attendance at the public schools on the day of survey; therefore, the absence of students due to serious bullying and/or serious family problems could have affected the results.

Several implications can be provided by this study. First, for bullying involvement among adolescents, CVA existence should be considered and an educational intervention for parents may be worth implementing. Second, such interventions should be considered for high school and junior high school students. Third, educational/health professionals should pay attention to the existence of current suicidal feelings of adolescents when they find students who are involved in bullying or experience CVA, and especially the combination thereof. Fourth, parents should be informed of the association of CVA with both bullying involvement and current suicidal feelings among adolescents.

In future studies, three analyses should be noted. First, causality between CVA and bullying involvement should be clarified using longitudinal data. Second, the frequency of CVA and bullying involvement should be assessed to reveal the potential dose-response relationship. Third, since cyberbullying is also associated with an increased risk of poor mental health [37,39], the association between CVA and cyberbullying should be addressed.

## Conclusions

CVA was associated with increased odds of bullying involvement in general adolescents. Furthermore, positive additive effects of CVA and all three types of bullying involvement on current suicidal feelings were found. Although correlational, this study suggested that avoidance of CVA might prevent bullying involvement and suicidal feelings in adolescents. In order to prevent suicidal feelings, health professionals should note that adolescent who are involved in bullying might be more likely to suffer from CVA. Future longitudinal study is required to reveal the causal relationship between CVA and bullying involvement. The frequency and severity of CVA and bullying involvement should also be assessed to examine the dose-response relationship.

## References

[1] Solberg ME, Olweus D. Prevalence estimation of school bullying with the Olweus Bully/Victim Questionnaire. Aggress Behav. 2003;29:239–68. 10.1002/ab.10047

[2] Craig W, Harel-Fisch Y, Fogel-Grinvald H, Dostaler S, Hetland J, Simons-Morton B, et al A cross-national profile of bullying and victimization among adolescents in 40 countries. Int J Public Health. 2009;54:216–24. 10.1007/s00038-009-5413-9 19623475 PMC2747624

[3] Reijntjes A, Kamphuis JH, Prinzie P, Telch MJ. Peer victimization and internalizing problems in children: a meta-analysis of longitudinal studies. Child Abus Negl. 2010;34:244–52. 10.1016/j.chiabu.2009.07.009 20304490

[4] Bond L, Carlin JB, Thomas L, Rubin K, Patton G. Does bullying cause emotional problems? A prospective study of young teenagers. BMJ (Clinical research ed.). 2001;323(7311):480–4. 10.1136/bmj.323.7311.480 11532838 PMC48131

[5] Copeland WE, Wolke D, Angold A, Costello EJ. Adult psychiatric outcomes of bullying and being bullied by peers in childhood and adolescence. JAMA psychiatry. 2013;70:419–26. 10.1001/jamapsychiatry.2013.504 23426798 PMC3618584

[6] Ttofi MM, Farrington DP, Losel F, Loeber R. Do the victims of school bullies tend to become depressed later in life? A systematic review and meta-analysis of longitudinal studies. J Aggress Confl Peace Res. 2011;3:63–73. 10.1108/17596591111132873

[7] Winsper C, Lereya T, Zanarini M, Wolke D. Involvement in bullying and suicide-related behavior at 11 years: a prospective birth cohort study. J Am Acad Child Adolesc Psychiatry. 2012;51:271–82. 10.1016/j.jaac.2012.01.001 22365463

[8] Herba CM, Ferdinand RF, Stijnen T, Veenstra R, Oldehinkel AJ, Ormel J, et al Victimisation and suicide ideation in the TRAILS study: specific vulnerabilities of victims. J Child Psychol Psychiatry Allied Discip. 2008;49:867–76. 10.1111/j.1469-7610.2008.01900.x 18492041

[9] Klomek AB, Kleinman M, Altschuler E, Marrocco F, Amakawa L, Gould MS. High school bullying as a risk for later depression and suicidality. Suicide Life-Threatening Behav. 2011;41:501–16. 10.1111/j.1943-278X.2011.00046.x 21793875 PMC3188679

[10] Klomek AB, Sourander A, Gould M. The association of suicide and bullying in childhood to young adulthood: a review of cross-sectional and longitudinal research findings. Canadian Journal of Psychiatry. 2010 282–8. 10.1016/j.ypsy.2010.10.092 20482954

[11] Klomek AB, Sourander A, Niemelä S, Kumpulainen K, Piha J, Tamminen T, et al Childhood bullying behaviors as a risk for suicide attempts and completed suicides: a population-based birth cohort study. J Am Acad Child Adolesc Psychiatry. 2009;48:254–61. 10.1097/CHI.0b013e318196b91f 19169159

[12] Arseneault L, Bowes L, Shakoor S. Bullying victimization in youths and mental health problems: “much ado about nothing”? Psychol Med. 2010;40:717–29. 10.1017/S0033291709991383 19785920

[13] Takizawa R, Maughan B, Arseneault L. Adult health outcomes of childhood bullying victimization: evidence from a five-decade longitudinal British birth cohort. Am J Psychiatry. 2014;171:777–84. 10.1176/appi.ajp.2014.13101401 24743774

[14] Vreeman RC, Carroll AE. A systematic review of school-based interventions to prevent bullying. Arch Pediatr Adolesc Med. 2007;161:78–88. 10.1016/S0084-3970(08)70648-0 17199071

[15] Olweus D, Limber SP. Bullying in school: evaluation and dissemination of the olweus bullying prevention program. Am J Orthopsychiatry. 2010;80:124–34. 10.1111/j.1939-0025.2010.01015.x 20397997

[16] Bowes L, Arseneault L, Maughan B, Taylor A, Caspi A, Moffitt TE. School, neighborhood, and family factors are associated with children’s bullying involvement: a nationally representative longitudinal study. J Am Acad Child Adolesc Psychiatry. 2009;48:545–53. 10.1097/CHI.0b013e31819cb017 19325496 PMC4231780

[17] Lereya ST, Samara M, Wolke D. Parenting behavior and the risk of becoming a victim and a bully/victim: a meta-analysis study. Child Abus Negl. Elsevier Ltd; 2013;37:1091–108. 10.1016/j.chiabu.2013.03.001 23623619

[18] Shetgiri R, Lin H, Flores G. Trends in risk and protective factors for child bullying perpetration in the United States. Child Psychiatry Hum Dev. 2013;44:89–104. 10.1007/s10578-012-0312-3 22661150

[19] Schwartz D, Dodge KA, Pettit GS, Bates JE. Friendship as a moderating factor in the pathway between early harsh home environment and later victimization in the peer group. The Conduct Problems Prevention Research Group. Dev Psychol. 2000;36:646–62. 10.1037/0012-1649.36.5.646 10976604 PMC2767178

[20] Schiamberg L, Barboza G, Chee G, and Hsieh M. The adolescent-parent context and positive youth development in the ecology of cyberbullying in a social-ecological approach to cyberbullying. Wright MF, editor. 2016.

[21] McKenna M., Hawk E., Mullen J., & Hertz M. Bullying among middle school and high school students-Massachusetts, 2009 (Reprinted from MMWR, vol 60, pg 465–71, 2011). JAMA-JOURNAL Am Med Assoc. 2011;305(22): 2283–6. 21508922

[22] Tochigi M, Nishida A, Shimodera S, Oshima N, Inoue K, Okazaki Y, et al Irregular bedtime and nocturnal cellular phone usage as risk factors for being involved in bullying: a cross-sectional survey of Japanese adolescents. PLoS One. 2012;7 e45736 10.1371/journal.pone.0045736 23029211 PMC3446940

[23] Ando S, Yamasaki S, Shimodera S, Sasaki T, Oshima N, Furukawa T a, et al A greater number of somatic pain sites is associated with poor mental health in adolescents: a cross-sectional study. BMC Psychiatry. 2013;13:30 10.1186/1471-244X-13-30 23327684 PMC3598352

[24] Nishida A, Shimodera S, Sasaki T, Richards M, Hatch SL, Yamasaki S, et al Risk for suicidal problems in poor-help-seeking adolescents with psychotic-like experiences: findings from a cross-sectional survey of 16,131 adolescents. Schizophr Res. 2014;159:257–62. 10.1016/j.schres.2014.09.030 25315221

[25] School Basic Survey; Ministry of Education, Culture, Sports, Science and Technology. 2014.

[26] Population census of Japan; Ministry of Internal Affairs and Communications. 2010.

[27] Paykel ES, Myers JK, Lindenthal JJ, Tanner J. Suicidal feelings in the general population: A prevalence study. Br J Psychiatry. 1974;124(5):460–9. 10.1192/bjp.124.5.460 4836376

[28] Nishida A, Sasaki T, Nishimura Y, Tanii H, Hara N, Inoue K, et al Psychotic-like experiences are associated with suicidal feelings and deliberate self-harm behaviors in adolescents aged 12–15 years. Acta Psychiatr Scand. 2010;121(4):301–7. 10.1111/j.1600-0447.2009.01439.x 19614622

[29] Nansel TR, Overpeck M, Pilla RS, Ruan WJ, Simons-Morton B, Scheidt P. Bullying behaviors among US youth: prevalence and association with psychosocial adjustment. JAMA. 2001;285:2094–100. 10.1001/jama.285.16.2094 11311098 PMC2435211

[30] Spriggs AL, Iannotti RJ, Nansel TR, Haynie DL. Adolescent bullying involvement and perceived family, peer and school relations: commonalities and differences across race/ethnicity. J Adolesc Heal. 2007;41:283–93. 10.1016/j.jadohealth.2007.04.009 17707299 PMC1989108

[31] Bandura A. Social learning theory. Social Learning Theory. 1971;1–46.

[32] McMurran M, Blair M, Egan V. An investigation of the correlations between aggression, impulsiveness, social problem-solving, and alcohol use. Aggress Behav. 2002;28:439–45. 10.1002/ab.80017

[33] Jacobson KC, Prescott CA, Kendler KS. Sex differences in the genetic and environmental influences on the development of antisocial behavior. Dev Psychopathol. 2002;14:395–416. 10.1017/S0954579402002110 12030698

[34] Rhee SH, Waldman ID. Genetic and environmental influences on antisocial behavior: a meta-analysis of twin and adoption studies. Psychol Bull. 2002;128:490–529. 10.1037/0033-2909.128.3.490 12002699

[35] Smokowski PR, Kopasz KH. Bullying in School: an overview of types, effects, family characteristics, and intervention strategies. Natl Assoc Soc Work. 2005;17:101–10. 10.1093/cs/27.2.101

[36] Silverman AB, Reinherz HZ, Giaconia RM. The long-term sequelae of child and adolescent abuse: a longitudinal community study. Child Abus Negl. 1996;20:709–23. 10.1016/0145-2134(96)00059-2 8866117

[37] Hinduja S, Patchin JW. Bullying, cyberbullying, and suicide. Arch Suicide Res. 2010;14(3):206–21. 10.1080/13811118.2010.494133 20658375

[38] Kaltiala-Heino R, Rimpelä M, Marttunen M, Rimpelä A, Rantanen P. Bullying, depression, and suicidal ideation in Finnish adolescents: school survey. BMJ. 1999;319(7206):348–51. 10.1136/bmj.319.7206.348 10435954 PMC28187

[39] Juvonen J, Gross EF. Extending the school grounds?—Bullying experiences in cyberspace. J Sch Health. 2008;78(9):496–505. 10.1111/j.1746-1561.2008.00335.x 18786042

